# Supplementary material for: A combined variational and diagrammatic quantum Monte Carlo approach to the many-electron problem
Source: Nat Commun. 2019 Aug 19;10:3725. doi: 10.1038/s41467-019-11708-6 (PMC6700185; doi:10.1038/s41467-019-11708-6)
Supplement: Supplementary file 1 — Supplementary Information [file 41467_2019_11708_MOESM1_ESM.pdf]

# Supplementary Material

Kun Chen* and Kristjan Haule†
*Department of Physics and Astronomy, Rutgers University, Piscataway, New Jersey 08854, USA*
(Dated: July 16, 2019)

### Supplementary Note 1: Conserving diagrammatic expansion

This section introduces two conserving diagrammatic techniques, which are called CFS and VCCFS in the main text, to calculate the polarization $P$ (or susceptibility $\chi$). Both schemes preserve the exact crossing symmetry and conservation laws (particle number, momentum, energy, etc.) order by order. We note that the particle-number conservation law of the polarization $P(\mathbf{q} \to 0, \tau) \to const$ is essential for the Coulomb electron gas, in order to properly describe the plasmon physics.

The conserving diagrammatic expansions for the polarization can be constructed with the Baym-Kadanoff approach [1, 2], which is briefly reviewed below, before presenting the computational schemes used in the main text. In the Baym-Kadanoff approach one first introduces an external potential coupled to the density operator of the system,

$$S[\psi^\dagger, \psi; U] = S[\psi^\dagger, \psi] - \int d1d2\ \psi^\dagger(1)U(1,2)\psi(2), \quad (1)$$

where $\psi$ are a Grassmann field for the electrons; the indexes represent spatial, temporal and spin variables. The generating functional for the connected correlation functions is defined as $\ln Z[U]$ with,

$$Z[U] = \int \mathscr{D}\psi^\dagger \mathscr{D}\psi e^{-S[\psi^\dagger, \psi; U]}. \quad (2)$$

For a given approximation to $\ln Z[U]$, one can derive a conserving approximation for the one-particle Green's function by making sure that

$$G(1,1') = \left.\frac{\delta \ln Z[U]}{\delta U(1',1)}\right|_{U\to 0}, \quad (3)$$

while the two particle correlation function (charge, or spin correlation function if spin indexes are not summed), should satisfy

$$\chi(1,2) = \left.\frac{\delta G(2,2^+;U)}{\delta U(1^+,1)}\right|_{U\to 0}, \quad (4)$$

where the notation $1^+$ and $2^+$ indicates the time ordering of the field operators. The polarization, for which we will develop a diagrammatic expansion, is related to the correlation function $\chi$ by

$$\chi(1,2) = -P(1,2) + \int d3d3' P(1,3)v_{bare}(3,3')\chi(3',2), \quad (5)$$

where $v_{bare}$ is the *unscreened* Coulomb interaction. Note that the second term vanishes for the spin correlation function $\chi_{zz}$ in the unpolarized electron gas.

We will apply the above algorithm to the uniform electron gas model defined by the Lagrangian $L = L_0 + \Delta L$, where the solvable part is

$$L_0 = \sum_{\mathbf{k}\sigma} \psi^\dagger_{\mathbf{k}\sigma}\left(\frac{\partial}{\partial\tau} - \mu + \mathbf{k}^2 + v_{\mathbf{k}}(\xi=1)\right)\psi_{\mathbf{k}\sigma} \quad (6)$$
$$+ \sum_{\mathbf{q}\neq 0} \phi_{-\mathbf{q}} \frac{q^2+\lambda_{\mathbf{q}}}{8\pi}\phi_{\mathbf{q}},$$

and the correction is

$$\Delta L = -\sum_{\mathbf{k}\sigma} \psi^\dagger_{\mathbf{k}\sigma} v_{\mathbf{k}}(\xi)\psi_{\mathbf{k}\sigma} - \xi \sum_{\mathbf{q}\neq 0}\phi_{-\mathbf{q}}\frac{\lambda_{\mathbf{q}}}{8\pi}\phi_{\mathbf{q}} \quad (7)$$
$$+ \sqrt{\xi}\frac{i}{\sqrt{2V}}\sum_{\mathbf{q}\neq 0}\left(\phi_{\mathbf{q}}\rho_{-\mathbf{q}} + \rho_{\mathbf{q}}\phi_{-\mathbf{q}}\right). \quad (8)$$

This Lagrangian was introduced in the main part of the text. Here the density $\rho$ is $\rho_{\mathbf{q}} = \sum_{\mathbf{k}\sigma}\psi^\dagger_{\mathbf{k}\sigma}\psi_{\mathbf{k}+q\sigma}$. Note that the effective potential $v_{\mathbf{k}}$ and the inverse screening length $\lambda$ in $L_0$ are compensated by the counter-terms in the correction $\Delta L$. The parameter $\xi$ is set to unity at the end of the calculation.

In the Baym-Kadanoff approach the external potential term $U(1,2)$ should be added to the solvable part $L_0$, and then the perturbative expansion for the generating functional $\ln Z[U]$ should be carried out using the standard Feynman diagrammatic expansion with building blocks shown in Figure 1. Note that the diagrammatic series constructed in this way only implicitly depends on the external potential $U$ through the bare electron propagator $g[U]^{-1} = -\frac{\partial}{\partial\tau} + \mu - \mathbf{k}^2 - v_{\mathbf{k}} + U$.

Now we are ready to discuss the Feynman diagrammatic expansion used in our work. We will first discuss the CFS scheme. To do this, we generate all free energy diagrams of order $N-1$, for example the diagram in Figure 1 of the main text, where the effective potential $v_{\mathbf{k}}$ is regarded as an arbitrary function, independent of $U$. We then calculate the two-particle correlation function with the second derivatives with respect to external potential $U$,

$$\chi(1,2) = \left[\frac{\delta^2 \ln Z[U]}{\delta U(1^+,1)\delta U(2^+,2)}\right]_{v_{\mathbf{k}}=\Sigma^x_{\mathbf{k}}+\cdots, U=0},$$

*Electronic address: chenkun0228@gmail.com
†Electronic address: haule@physics.rutgers.edu

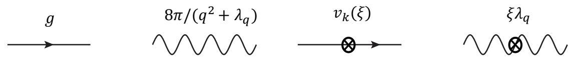


Supplementary Fig. 1: **Feynman diagram building blocks:** The bare electron propagator $g$ describes an electron propagating in an effective potential $v_{\mathbf{k}}$, the interaction line $8\pi/(q^2+\lambda_q)$ represents a bosonic propagator with an effective mass $\sim \lambda$, which makes the Coulomb repulsion short ranged. The counter terms, which compensate for our choice of the effective $L_0$, are proportional to $v_k(\xi)$ (the single-particle counter term) and $\xi\lambda_q$ (the interaction counter term), and are depicted in the last two diagrams.

Note that the $U$ derivative is taken by the chain rule, i.e., $\delta/\delta U = (\delta g/\delta U)(\delta/\delta g)$, where the the $U$-derivative of the propagator is simple: it just splits the propagator into two by inserting an external vertex,

$$\frac{\delta g(1,2;U)}{\delta U(3^+,3)} = -g(1,3)g(3,2). \tag{9}$$

This relation is derived by taking the derivative of the identity $g^{-1}g = 1$, which is $g^{-1}dg/dU + (dg^{-1}/dU)g = 0$, therefore $dg/dU = -g(dg^{-1}/dU)g$ and $dg^{-1}/dU = 1$, provided $v_{\mathbf{k}}$ is independent of $U$. Diagrammatically, a derivative $\delta/\delta U$ removes a single-particle propagator from the Feynman diagram $(\delta/\delta g)$, and we then replace it with an external vertex and the two propagators, i.e., $\delta g/\delta U = -gg$. In other words, it inserts an external vertex on an existing bare electron propagator. Note that this operation increases the diagram order by one. Finally, after the derivative is taken, we substitute $v_{\mathbf{k}}$ with its expression in terms of the exchange self-energy,

$$v_{\mathbf{k}} = \xi\,(\Sigma^x_{\mathbf{k}} - \Sigma^x_{k_{\rm F}}) + \xi^2\, s_2 + \xi^3\, s_3 \cdots . \tag{10}$$

With the above described algorithm, we obtain the conserving expansion for the two particle correlation function $\chi$, however, the convergence for the dielectric function is even faster when the expansion is carried out for the polarization function defined by Eq. 5. In the momentum and frequency space, the two are related by

$$\chi(\mathbf{q}) = -\frac{P_{\mathbf{q}}}{1 - P_{\mathbf{q}}\frac{8\pi}{\mathbf{q}^2}} \tag{11}$$

or $\chi(\mathbf{q}) = -[P_{\mathbf{q}} + P_{\mathbf{q}}\frac{8\pi}{\mathbf{q}^2}P_{\mathbf{q}} + P_{\mathbf{q}}\frac{8\pi}{\mathbf{q}^2}P_{\mathbf{q}}\frac{8\pi}{\mathbf{q}^2}P_{\mathbf{q}} + \cdots]$, meaning that $P_{\mathbf{q}}$ is the irreducible part of $\chi(\mathbf{q})$ with respect to cutting the interaction propagator $\frac{8\pi}{\mathbf{q}^2}$ Similarly, when working with the screened interaction $\frac{8\pi}{\mathbf{q}^2+\lambda}$, we can rewrite

$$\frac{8\pi}{\mathbf{q}^2} = \frac{8\pi}{\mathbf{q}^2+\lambda}\sum_{n=0}^{\infty}\left(\frac{\xi\lambda}{8\pi}\frac{8\pi}{\mathbf{q}^2+\lambda}\right)^n \tag{12}$$

and therefore

$$\chi(\mathbf{q}) = -\frac{P_{\mathbf{q}}}{1 - P_{\mathbf{q}}\frac{8\pi}{\mathbf{q}^2+\lambda}\sum_{n=0}^{\infty}\left(\frac{\xi\lambda}{8\pi}\frac{8\pi}{\mathbf{q}^2+\lambda}\right)^n}, \tag{13}$$

which shows that $P_{\mathbf{q}}$ is now the irreducible part of $\chi(\mathbf{q})$ with respect to cutting the interaction propagator $\frac{8\pi}{\mathbf{q}^2+\lambda}$ or any combination of interaction with counter terms of arbitrary order, i.e., $\frac{8\pi}{\mathbf{q}^2+\lambda}(\frac{\xi\lambda}{8\pi}\frac{8\pi}{\mathbf{q}^2+\lambda})^n$. The resulting polarization diagrammatic expansion is shown in Figure 2.

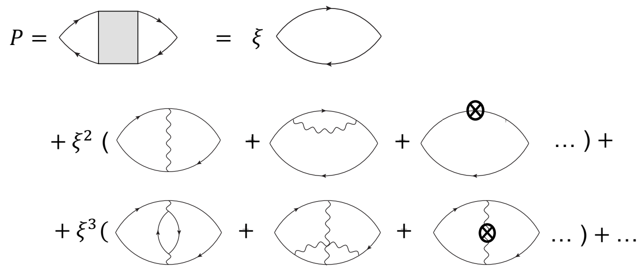


Supplementary Fig. 2: **Feynman diagrammatic expansion with counterterms:** The perturbative expansion for the polarization is formulated with the standard Feynman diagrams with counterterms. The shaded block represents all one-interaction-irreducible diagrams for the particle-hole four-point vertex function. Note that the single-particle counterterm first appears at the second order, while the interaction counter-term first appears at the third order. Note that in this work we choose the single-particle counterterm to be the negative Fock diagram contribution plus a chemical potential shift, therefore any diagram with a Fock sub-diagram insertion (such as the diagram three above) is exactly canceled by a counter-term (such as the diagram four), and the two can hence be removed. Consequently, one can simply drop the Fock sub-diagram insertion in the diagrammatic series, and keep only the chemical potential shift in the single-particle counter-term.

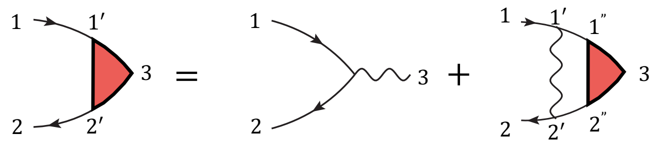


Supplementary Fig. 3: **Ladder-type vertex correction:** The ladder diagrams can be resummed by a Bethe-Salpeter equation.

In practice, we find that the electric charge renormalization, which correspondes to the three-leg-vertex correction in diagrams, becomes increasingly more important at the low density limit (with $r_{\rm s} \gtrsim 2$). Therefore, we introduce a vertex corrected scheme (VCCFS scheme), where we resume all the ladder-type diagrams.

The dressed ladder-type vertex correction can be calculated with a Bethe-Salpeter self-consistent equation, which is depicted in Figure 3. In each polarization diagram, we then replace the two bare external vertices with the dressed vertices (the three-leg-vertex). To avoid the double counting of the diagrams, we also eliminate all polarization diagrams which contains a ladder-type vertex

correction on either side of the diagram. This operation can be respresented by Figure 4, in which the power expansion in powers of $\xi$ automatically removes all diagrams with ladder-type vertex corrections on either end.

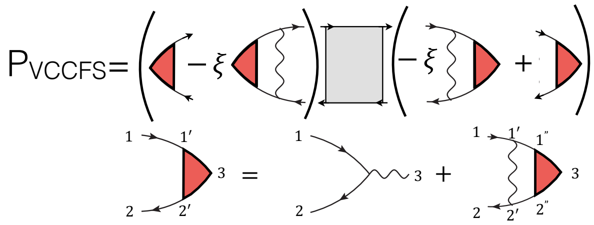

Supplementary Fig. 4: **VCCFS scheme for polarization diagrams:** The perturbative expansion for the polarization can be improved using the ladder resummation. The ladder vertex correction is attached to both sides (the left and right external vertex) and to all polarization diagrams. The double-counted diagrams are properly subtracted.

We emphasize here that all polarization diagrams in both schemes only involve the statically screened Coulomb interaction $\frac{8\pi}{\mathbf{q}^2+\lambda_\mathbf{q}}$. This is a nontrivial result, given that the definition of the polarization in Eq. (5) explicitly depends on the bare Coulomb interaction. Combing this feature with the fact that screened Coulomb interaction does not diverge in the long-wave-length limit, all polarization diagrams are now automatically regularized, making the Monte Carlo simulations much more efficient.

## Supplementary Note 2: Efficient Diagrammatic Monte Carlo Algorithm

In this section, we introduce a simple yet efficient Monte Carlo algorithm to evaluate high order Feynman diagrams. To calculate all order $N$ contributions, the diagrammatic Monte Carlo algorithm needs to integrate over all internal variables, such as momenta and times, and also sum over all topology of the diagrams, i.e.,

$$F_1^N = \int [d\tau]^{2N}[d\mathbf{k}]^{N+1} \sum_{topology} W[\{\tau\},\{\mathbf{k}\}] \tag{14}$$

All diagrams in the same order share the same set of interval variables. Due to the Fermi statistics, the sign of the integrand $W[\{\tau\},\{\mathbf{k}\}]$ alternates as the topology and internal variables change. However, a Monte Carlo algorithm can only handle positively defined weight functions. A straightforward choice is to sample the absolute value of the integrand $|W[\{\tau\},\{\mathbf{k}\}]|$, namely working with the sum,

$$F_3^N = \int [d\tau]^{2N}[d\mathbf{k}]^{N+1} \sum_{topology} |W[\{\tau\},\{\mathbf{k}\}]| \tag{15}$$

However, as pointed out by the previous studies [3], the sign cancellation between diagrams causes $F_1^N \ll F_3^N$. More specifically, although $F_3^N$ always diverge factorially with the number of diagrams, the series $F_1^N$ is much better behaved (diverging slowly, or even convergent if the series is within the convergence radius). This phenomenon is termed the "sign blessing" in Ref. [3]. As a result, the straightforward Monte Carlo scheme sampling $F_3^N$ to evaluate $F_1^N$ suffers from the notorious sign problem, and is very inefficient. In this work, we propose a Monte Carlo algorithm, which samples the following weight function,

$$F_2^N = \int [d\tau]^{2N}[d\mathbf{k}]^{N+1} |\sum_{topology} W[\{\tau\},\{\mathbf{k}\}]| \tag{16}$$

Thanks to the inequality $F_1^N \leq F_2^N \leq F_3^N$, a method sampling $F_2^N$ is guaranteed to suffer less sign problem, thus is more efficient than the straightforward approach. Of course, the efficiency of this approach relies on how small is $F_2^N$, and how close is $F_2^N$ to $F_1^N$. The minimization of $F_2^N$ can be achieved by optimizing the arrangement of interval variables of different diagrams, so that the sum of their weights with the same set of variables strongly cancel with each other. We will discuss this in more detail in the next section.

Now we summarize the main steps of the new diagrammatic Monte Carlo algorithm used in this work.

i) Write a script to generate all Feynman diagrams up to the desired truncation order (say order 6 in this work), including all necessary symmetry factors and counter-terms.

ii) Design an algorithm to properly assign interval variables to minimize the weight function $F_N^2$ (choice of basis). This algorithm will be described in the next section.

iii) Use the standard Metropolis algorithm to sample$F_N^2$ in order to calculate the high dimensional integral $F_N^1$. To properly normalize the integral $F_N^1$, we design an ansatz for a function, which can be integrated deterministically, and has parameters that can be adapted to the landscape of $F_N^2$.

Note that the Monte Carlo updates only need to randomly generate internal variables $\mathbf{k}$ and $\tau$, but do not need to change the diagram topology, so that the algorithm is extremely simple.

## Supplementary Note 3: "Sign-blessed" Group of Diagrams

In this section we explain the details of our algorithm to organize diagrams, so that an efficient diagrammatic Monte Carlo method can be implemented. We will show how the diagrams of a given order can be divided into

groups, where the diagrams in the same group are guaranteed to massively cancel with each other. The "sign-blessed" group may be obtained by grouping: i) diagrams that share the same set of internal variables, and those diagrams in which ii) the integrand $W[\{\tau\}, \{\mathbf{k}\}]$ massively compensate with each other. The first requirement is automatically satisfied for the connected diagrams of the same order $N$, as all order-$N$ connected diagram requires $N+1$ independent momentum/frequency variables or $2N$ space/time variables. The second requirement is much more challenging and can only be achieved by carefully examining the sign structure of the diagrams.

We identify two useful generic rules for the occurrence of the sign-blessing in fermionic systems with momentum-imaginary-time representation. One generic mechanism which is particularly important for fermions is the crossing symmetry, as depicted in Figure 5, namely permuting arbitrary two fermionic propagators causes an overall sign change to the diagram. If two fermionic propagators being exchanged carry similar momentum, which occurs near the Fermi surface, the direct and exchange diagrams strongly compensate with each other. It is therefore important to optimally arrange the internal variables so that the diagram integrand $W_{group}$ keeps the exact crossing symmetry.

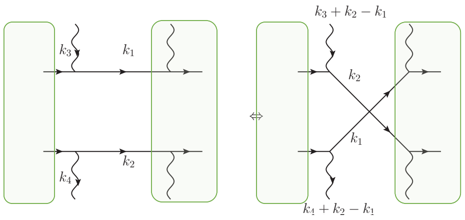


Supplementary Fig. 5: **Permutation using the crossing symmetry:** A permutation of a two fermionic propagators, as shown in the unshaded region of the figure, creates a new diagram with the opposite sign. If the incoming momentums/frequencies are similar, the two diagrams have almost the same absolute value and opposite sign, hence the sum of the two diagrams leads to "sign blessing". Under this operation, the rest of the diagram, together with the shaded regions, remains the same. In order to achieve the cancellation of the integrand (not just the resulting integral), the two diagrams have to be consistently labeled, and therefore the momentum/frequency labeling of the entire diagram outside the unshaded region has to be identical on the two diagrams.

Another generic mechanism is the conservation laws (or Ward identities). For example, the conserving diagrammatic expansions for the polarization proposed in the previous section satisfy $P(\mathbf{q} \to 0, \tau) \to 0$ when approaching the zero temperature. However, this is an emergent property satisfied only by the sum of a conserving group of diagrams. In fact, all individual polarization diagrams (except the bubble diagram) break the conservation law and fluctuate around zero. Therefore, we observe a strong sign cancellation between the diagrams in the same conserving group. According to the Baym-Kadanoff approach in Eq. (4), there is one-to-one correspondence between the minimal conserving groups for the polarization diagrams of the order $N$ and the $\ln Z$ diagrams of the order $N-1$ Indeed, for an arbitrary $\ln Z$ diagram, one can simply attach two external vertices to two of the bare electron propagators $g$ in all possible ways, and generate a conserving group for the polarization function. Strictly speaking, the sign blessing of the conserving groups is only guaranteed after integrating out all internal variables. However, provided that the internal variables of the polarization diagrams are inherited from the same free energy diagram, the operation of inserting two external vertices generates different time-ordered polarization diagrams, and leads to sign alternation within the conserving groups, implicitly encoding the sign blessing of the conservation law.

Now we are ready to propose the algorithm to group the diagrams and properly arrange internal variables. The algorithm is applicable to an arbitrary combination of momentum/frequency or space/time variables. To be consistent with the main text, we describe the algorithm with momentum/time representation. The main steps of the algorithm are:

i) Pick an arbitrary order-$N$ connected $\ln Z$ diagram, label all $2N$ time variables and choose $N+1$ *independent* momentum loops. Keep momentum loops as short as possible.

ii) Generate a new connected $\ln Z$ diagram by permuting two electron propagators, rearrange the momentum loops as described in Figure 5 so that they automatically form a complete and independent loop basis for the new diagram. Thanks to the crossing symmetry, the new diagram has the opposite sign to the starting diagram. This step is repeated until all $\ln Z$ diagrams are exhausted.

iii) For each $\ln Z$ diagram, attach two external vertices to two of the electron propagators in all possible ways, to generate a conserving group of polarization diagrams. The arrangement of the internal variables of the original $\ln Z$ should not be modified in this step, so that the generated polarization diagrams share common parts of the diagram (many equal propagators).

It is also possible to apply the above algorithm to Hugenholtz diagrams, which form a particular subset generated by the algorithm in Figure (5) (when the top and the bottom bosonic propagators are connected to each other). These diagrams combine the direct and exchange interaction into an antisymmetric four-point vertex. They are particularly convenient if one works with momentum/frequency, or momentum/time representation, and the interaction is instantaneous, as in our model Eq. (6) and Eq. (7).

Finally, we briefly discuss the benefits of grouping the diagrams in the diagrammatic Monte Carlo algorithm. There are two improvements in terms of the Monte Carlo efficiency. First, the total weight function $F_2^N$ sampled by the Markov chain is much smaller than $F_3^N$. This indicates the variance of the integrand is dramatically reduced, which improves the statistical error. Second, the

diagrams in the same group typically share many common objects (propagators and interactions). This simplifies the total diagram weight calculations in each Monte Carlo update. For example, all Feynman diagrams (up to $2^N$ diagrams at order $N$) that belong to the same Hugenholtz diagram, share the same set of propagators, thus they only need to be evaluated once. Indeed, all Feynman diagrams that belong to the same Hugelholtz diagram can be choosen to have all fermionic propagators identical. Those are computed once, and not $2^N$ times. Furthermore, the interaction lines are not identical, however, they contain a lot of common products. One can show that a binary tree can be constructed, with the depth equal to the number of Hugenholtz interaction propagators, in which each vertex of the binary tree adds either the direct or the exchange interaction to the Hugenholtz diagram. The leaves of such a binary tree contain exactly $2^N$ terms, corresponding to the products we need to evaluate the sum of $2^N$ Feynman diagrams, while the number of operations to evaluate such a tree grows as $O(N)$.


[1] Baym, G. and Kadanoff, L. P. Conservation Laws and Correlation Functions. Phys. Rev. **124**, 287299 (1961).

[2] Baym, G. Self-Consistent Approximations in Many-Body Systems. Phys. Rev. **127**, 13911401 (1962).

[3] Prokof'v, N. and Svistunov, B. Fermi-polaron problem: Diagrammatic Monte Carlo method for divergent sign-alternating series. Phys. Rev. B. **77**, 020408 (2008).
